# Increased renal corticomedullary FDG activity in a patient of NHL–malignant or benign?

**DOI:** 10.4103/0972-3919.78260

**Published:** 2010

**Authors:** Abhishek J Arora, Tushar Mohapatra, Sikandar Shaikh, Richa A Arora

**Affiliations:** Yashoda Hospital, Hyderabad, India

**Keywords:** FDG PET-CT, pyelonephritis, lymphoma

## Abstract

Authors describe diagnostic dilemma of differentiating pyelonephritis with lymphomatous involvement of kidney in a known case of lymphoma. FDG uptake pattern was non-discriminatory and pyelonephritis diagnosed retrospectively on follow up study. Authors emphasize the importance of recognition of features and subtle clues of infection evident on CT component of PET-CT.

Kidneys being the physiological route of excretion of F18-FDG pose significant problem while interpreting its involvement by focal or diffuse hypermetabolic lesions like malignancies and inflammatory processes. Physiological pelvicalyceal activity can be dealt with the help of intravenous diuretics, oral hydration, and delayed imaging. It is known that lesions of lymphoma with renal involvement are FDG avid and early identification of this pathology causing ARF is crucial for recovery of kidney function.[1,2] To differentiate the lymphomatous involvement of kidney from other inflammatory pathologies, due consideration is to be given to other CT features like renal calculi, perirenal fat stranding, and thickened perirenal fascia [Figure 1a–b].[3]

**Figure 1:**
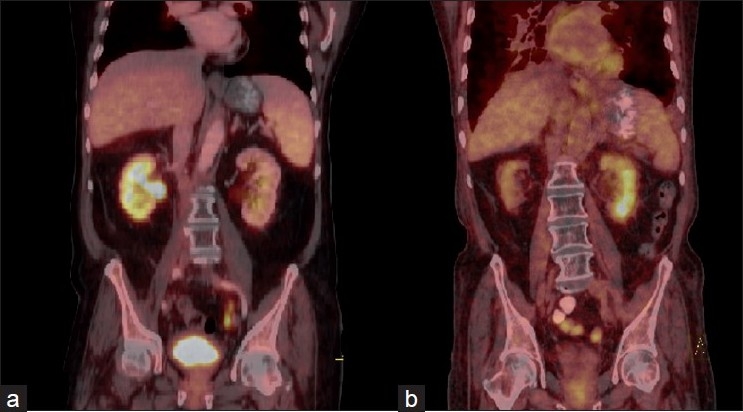
Review of pre- and post-therapy PET CT studies of a 71-year-old male patient, a known case of abdominal non–Hodgkin’s lymphoma, revealed significant reduction in size and metabolic activity of retroperitoneal lymphomatous lesion. Increased FDG activity in the corticomedullary space of right kidney in pretherapy study (a), reported as lymphomatous involvement, was not seen in the subsequent scan which showed decreased size. Serum creatinine levels showed increase in values from 0.8 to 2.3 during the interval between these studies, for which a non-contrast follow-up PET/CT study was done.- In follow-up PET/CT study, previously normal appearing left kidney showed intense corticomedullary tracer activity along with associated surrounding fat stranding and thickened perirenal fascia bilaterally (b). Above findings suggested inflammatory/pyelonephritic nature of the disease, thus impressing upon the importance of early detection of inflammatory etiology of the kidney and differentiating it from malignant involvement while reporting PET/CT cases showing increased corticomedullary renal uptake.
